# Physiology of salt tolerance introgressions from *Solanum galapagense* in the domesticated tomato

**DOI:** 10.3389/fpls.2025.1568851

**Published:** 2025-05-15

**Authors:** Maria-Sole Bonarota, Sean Fenstemaker, Hans Vasquez-Gross, Juli Petereit, Patricia Santos, David M. Francis, Felipe H. Barrios-Masias

**Affiliations:** ^1^ Department of Agriculture, Veterinary, and Rangeland Sciences. University of Nevada, Reno, NV, United States; ^2^ Department of Horticulture and Crop Science. The Ohio State University, Columbus, OH, United States; ^3^ Nevada Bioinformatics Center (RRID: SCR_017802), University of Nevada, Reno, NV, United States

**Keywords:** RNA-seq, gas exchange, root hydraulic conductance, photosynthesis, salinity stress

## Abstract

**Introduction:**

Wild trait introgression is a valuable breeding tool for increasing tomato salinity tolerance. However, this process often results in deleterious linkage drag. Understanding the physiological and molecular mechanisms underlying salinity response can aid in developing salt-tolerant cultivars while minimizing undesirable traits. This study investigates the salinity response of the tomato cultivar OH8245, *Solanum galapagense* accession LA1141, and two derived introgression lines (ILs SG18_197 and SG18_247) that were previously screened for salt tolerance traits.

**Methods:**

The physiological and molecular responses of OH8245, LA1141, and the two ILs were analyzed under salinity stress. Key salinity tolerance traits were evaluated, including root characteristics, water status, ion homeostasis, stomatal density, photosynthetic rate, and relative growth rate. Differential gene expression analysis was conducted to identify genes associated with salinity tolerance, comparing the number and uniqueness of differentially expressed genes (DEGs) across genotypes.

**Results:**

*S. galapagense* LA1141 exhibited multiple salinity tolerance traits, such as higher specific root length, increased root hydraulic conductivity, and improved plant water status. It also maintained better ion homeostasis and had lower stomatal density compared to OH8245. In contrast, OH8245 demonstrated traits supporting greater biomass accumulation, including a higher photosynthetic rate and relative growth rate. Differential gene expression analysis revealed that LA1141 had the fewest DEGs (706), whereas OH8245 had one of the highest (2524), suggesting a constitutive set of genes contributing to salinity or abiotic stress tolerance. Additionally, 40 DEGs were uniquely found in LA1141 under salinity, with nine and 16 of these transferred to ILs SG18_197 and SG18_247, respectively.

**Discussion:**

Salinity tolerance is a complex trait that imposes an energy cost on the plant. However, key beneficial traits, including improved plant water potential, higher photosynthetic rate, and a lower sodium/potassium ratio, were successfully transferred from LA1141 to at least one of the ILs. These findings provide valuable insights for tomato breeding programs aimed at enhancing salinity tolerance while balancing growth and stress resistance traits.

## Introduction

Soil salinity, defined as an electrical conductivity (EC) >2.5 dS m^-1^, affects 20% of cultivated lands and is one of the most challenging environmental conditions that limit crop yield (Machado and Serralheiro, 2017; FAO, 2021). High-salinity affected areas are expanding at a 10% rate per year due to poor-quality irrigation water, high evapotranspiration rates and inappropriate use of fertilizers, especially in arid and semi-arid regions (Liu et al., 2020). Salts can be leached away with the use of high-quality water (i.e., low EC), but this method has become unsustainable and impractical (Boretti and Rosa, 2019). Thus, improving crop productivity under salinity stress can help reduce food insecurity under the increasingly water-limited and saline conditions of the future (Masson-Delmotte et al., 2019). Addressing salt tolerance in crops is challenging because soil salinity causes physical (disturbed soil aggregates and lower soil water potential) (Barzegar et al., 1994; Sheldon et al., 2017), chemical (nutrient imbalance and ion toxicity) (Grattan and Grieve, 1998), and biological (altered soil and rhizosphere microbiome) (Santos et al., 2021) stresses to the plant.

Tomato (*Solanum lycopersicum*) productivity decreases 10% per unit increase of EC after the threshold of 2.5 dS m^-1^ (Saranga et al., 1991). Tomato wild relatives (*Lycopersicon* clade) are a valuable genetic resource for salinity tolerance (Bonarota et al., 2022), and advances in breeding technologies such as quantitative trait loci (QTL) mapping, genome-wide association studies (GWAS), the use of linked markers in selection and of introgression lines has enabled the widespread use of wild tomato species for breeding purposes (for an extensive review, see Labate et al., 2007 and Chaudhary et al., 2019). Higher water and nutrient uptake capacity, ion balance (Albaladejo et al., 2017; Kashyap et al., 2020), antioxidant activity (Frary et al., 2010), hormonal signaling (Gharbi et al., 2017), and relative growth rate (Pailles et al., 2020) are some of the most important salt tolerance responses found in tomato wild relatives. However, the introgression of traits from wild relatives to elite cultivars remains challenging due to reproductive barriers and linkage drag (Swarup et al., 2021). More importantly, the complexity of a plant’s salt tolerance response involves the interaction of a suite of traits, which may inadvertently include tradeoffs such as reductions in biomass accumulation; thus, understanding these tradeoffs to successfully transfer complex salt tolerance mechanisms from wild relatives to the cultivated tomato is necessary.

*Solanum galapagense* is a close wild relative of the domesticated tomato that can be reciprocally hybridized with it (Rick, 1956), and is known for its salt tolerance (Taylor, 1986; Grandillo et al., 2011; Pailles et al., 2020). The *S. galapagense* accession LA1141 has been utilized for its purple fruit color and its drought tolerance (Fenstemaker et al., 2022a, 2022b). The present study aimed to characterize the salinity response of *S. galapagense* accession LA1141 (herein referred to as LA1141) and identify potential traits that could be used to improve plant water relations and ion balance under salinity in the cultivated tomato. We evaluated two introgression lines (ILs; SG18_197 and SG18_247), selected based on their higher plant water status under salinity stress (**Supplementary Figure S1**), and derived from LA1141 and the processing tomato variety OH8245 (*S. lycopersicum* L.).

## Materials and methods

### Plant material and experimental design

Four genotypes were used in this study: *S. galapagense* LA1141, *S. lycopersicum* OH8245 (Berry et al., 1991), and two ILs derived from them (SG18_197 and SG18_247) (Fenstemaker et al., 2022b). The two ILs were selected from a population subsample of ten ILs, based on morpho-physiological responses such as better plant water status and higher photosynthetic rate under salinity (**Supplementary Figure S1**). Four trials were conducted between Summer 2021 and Summer 2023 at the Valley Road Greenhouse Complex (University of Nevada, Reno). The seeds were germinated at 28°C and transferred to Conviron chambers (photoperiod: 14 h; temperature: 24°C to 28.5°C; relative humidity of 20 to 30%) in 72-well trays. After four weeks, seedlings were transplanted into square pots (7-cm wide and 23-cm tall; Stuewe & Sons, Inc., Oregon, USA) filled with 1.5 cm-layer of fritted clay at the bottom and 20 cm-layer of sand on top. In the greenhouse, temperatures were between 28°C and 24°C (day and night), photoperiod was 14 h, and relative humidity was between 20% and 30%. After transplanting, plants were allowed to establish for seven days. Plants (three to four leaf stage) were assigned to a control (CTR = 0 mM NaCl and 0 mM CaCl_2_; EC =1.5 dS m^-1^) and salinity treatment (SAL = 60 mM NaCl and 30 mM CaCl_2_; EC =12 dS m^-1^). Experiments were performed in a randomized complete block design (RCBD) with eight plants per block (i.e., four genotypes and two salinity treatments). Twelve biological replicates were used for physiological characterization in each trial (96 plants per trial) and three biological replicates from the second trial were used for RNA-seq and SNP analysis (24 total plants).

### Plant biomass

Plants were photographed every three days (total of seven photos) to determine relative growth rate (RGR) and plant height. Images were analyzed using the ImageJ (Fiji software). RGR (cm^2^ day^-1^) was the slope (R^2^ >0.90) between plant frontal area and day of the experiment. After 21 to 28 days of treatment (DOT), roots, stems and leaves were separately oven-dried at 60°C and dry weight (DW) recorded for each plant organ.

### Root physiology

Root hydrostatic and osmotic hydraulic conductivity (*Lp*_h__yd_ and *Lp*_os_) were evaluated in intact root systems as in Barrios-Masias et al. (2015). Plants were harvested after 21 to 28 DOT, when roots had intact root tips and before reaching the bottom of the pot, which results in damage and lateral branching. For the calculation of root hydraulic conductivity (*Lp*_r_), measurements were normalized using fresh root biomass instead of root surface area and denoted with “*” (Hernandez-Espinoza and Barrios-Masias, 2020).

After ten to 14 DOT, roots were washed, scanned using an Epson scanner (Perfection V700/V750 2.80A, Place) set to 400 dpi, and analyzed using WinRHIZO (version 4.0b, Regent Instruments Inc., Quebec, Canada). The roots were oven-dried and weighed. Specific root length (SRL; cm g^-1^) was calculated as: root length/root DW. Root tissue density (RTD; g cm^-3^) was calculated as: root DW/root volume. The same root scans were used to analyze root diameter classes, which were merged into roots with diameter >1 mm and roots with diameter <1 mm.

### Leaf physiology and water potential

Between the eighth and 14^th^ DOT, leaf gas exchange was measured on a fully developed leaflet between 1030 and 1200 h using a portable gas-exchange system (LiCOR model 6400XT, Nebraska, USA) set at 400 μmol s^−1^ flow rate, 400 μmol mol^−1^ reference CO_2_, 25°C block temperature and 1800 μmol m^−2^ s^−1^ PAR. Data were recorded after reaching photosynthetic rate (P_n_) and stomatal conductance (g_s_) stability. Night respiration was measured between 2200 and 2400 h on the same leaflet as P_n_ and g_s_, and with the same LiCOR-6400XT and settings, except that flow rate was set at 200 μmol s^−1^ and no light provided (i.e., PAR = 0 μmol m^−2^ s^−1^).

Specific leaf area (SLA; cm^2^ g^-1^) was determined after 20 DOT from a scanned leaflet using ImageJ for area and DW after oven-drying at 60°C. Stomatal morphology (stomatal length and guard cell width) and density were determined on a 1-mm^2^ area from abaxial leaflet imprints (Spence et al., 1986) from four time points within ten DOT using ImageJ. At the same time points, leaf abscisic acid (ABA) concentration was determined as described in Hernandez-Espinoza and Barrios-Masias (2020) and following the manufacturer instructions of the ABA ELISA kit (Cusabio Biotechnology Co., Ltd, Wuhan, China). For nutrient analysis, fully developed leaflets without petioles were harvested after 21 DOT, dried at 60°C for 48 h, and digested using the protocol from Miller (1998). After digestion, leaf nutrient content (Ca, Cu, Mg, Mn, K, and Na) was quantified using a microwave plasma-atomic emission spectrometer (Agilent 4210, California, USA) at the UNR Core Analytical Laboratory. Leaf carbon (C), nitrogen (N) and δ^13^C were quantified as in Bristow et al. (2021).

Stem water potential (Ψ_stem_) was measured at ~1200 h using a Scholander pressure chamber (PMS Instrument; model 1505D), and leaf osmotic potential (Ψ_π_) determined after 20 DOT from a mature leaf from the top of the canopy (Bonarota et al., 2024a).

### RNA-seq and SNP analysis

After 21 DOT, the bottom 5-cm from the root tips (three biological replicates) were excised and triple washed with DI water, stored in RNAse free 50-ml Falcon tubes (USA Scientific, Ocala, FL, USA), frozen in liquid N within ~10 min, and stored at -80°C until processing. Total RNA was extracted using the Spectrum™ Plant Total RNA Kit (Sigma-Aldrich, Saint Louis, MO, USA), in-column DNAse treated, purified and concentrated using the RNA Clean and Concentrator™ (Zymo Research, Irvine, CA, USA). All samples had an A_260/280_ ≥1.8 and an RNA Integrity Number ≥8. Sequencing of cDNA libraries was done with Illumina NextSeq 2000 (Illumina, USA) platform. Quality control of the reads (100 base pairs, paired end) was performed with FastQC v0.11.9 before and after trimming, and a unified multi-sample report was generated using MultiQC v1.12. Trimming and filtering was conducted with fastp v0.20 with default parameters. After trimming, RSEQC v4.0 package was used to perform quality control for read GC content and read duplication. To identify possible regions of LA1141 that did not align to the tomato reference genome Sl4.0, we aligned the reads to different non-reference genomes (*S. lycopersicum* OH8245, S. pimpinellifolium LA2093 and LA1670), and we decided to continue using the tomato reference genome Sl4.0 because of higher percent of proper pairs and assigned reads (**Supplementary Table S1**). Using STAR v2.7, the reads were aligned to the tomato reference genome version Sl4.0 and annotation ITAG4.1. The differentially expressed genes (DEGs) in salinity versus control treatment were identified for each genotype using DESeq2 Bioconductor package (Love et al., 2014). DEGs were defined as false discovery rate (FDR) adjusted *p* value <0.05 and |log_2_FC| >2. Gene ontology enrichment analysis was performed with PANTHER v18.0.

The expression levels of eight genes were analyzed with RT-PCR to validate the RNA-seq results, and primer sequences are listed in **Supplementary Table S2**. The analysis included three replicates and was performed as in Bonarota et al. (2024a).

Single nucleotide polymorphisms (SNPs) on the RNA-seq data were identified using freebayes v1.3.6 (Garrison and Marth, 2012) for each genotype against the tomato reference Sl4.0. The resulting VCF file was filtered with the “VCFfilter” tool, part of the VCFlib module (Garrison et al., 2021), to remove all SNPs with a Phred score <25. Only homozygous SNPs were kept for further analysis. The impact of the SNPs was predicted with SnpEff v5.0 (Cingolani et al., 2012).

### Statistical analysis

Statistical analysis was conducted in R 4.3.1 (R Core Team, 2022). The effects of “genotype”, “treatment”, and their interaction on the response variables were analyzed with linear mixed-effects models using lmer function (lme4 package; Bates et al., 2015), followed by Anova function (car package; Fox and Weisberg, 2019) to get the p-values for the main effects. The random effects were selected using Akaike information criterion (AIC) between trial, block within trial, and day of measurement in repeated measures analysis (e.g., leaf gas exchange data). The QQ plot for normal distribution and boxplots for homogeneity of variance were used to test that data fulfilled the ANOVA assumptions on the residuals of the model. For all models, the α for the main effects was set at 0.05 level. When the calculated p-value was lower than the chosen α, the null hypothesis was rejected and pairwise comparison for multiple testing was conducted using the emmeans function (emmeans package; Lenth, 2023), using unrestricted least significant difference (LSD) test (Saville, 2015).

## Results

### Root traits

**Figure 1:**
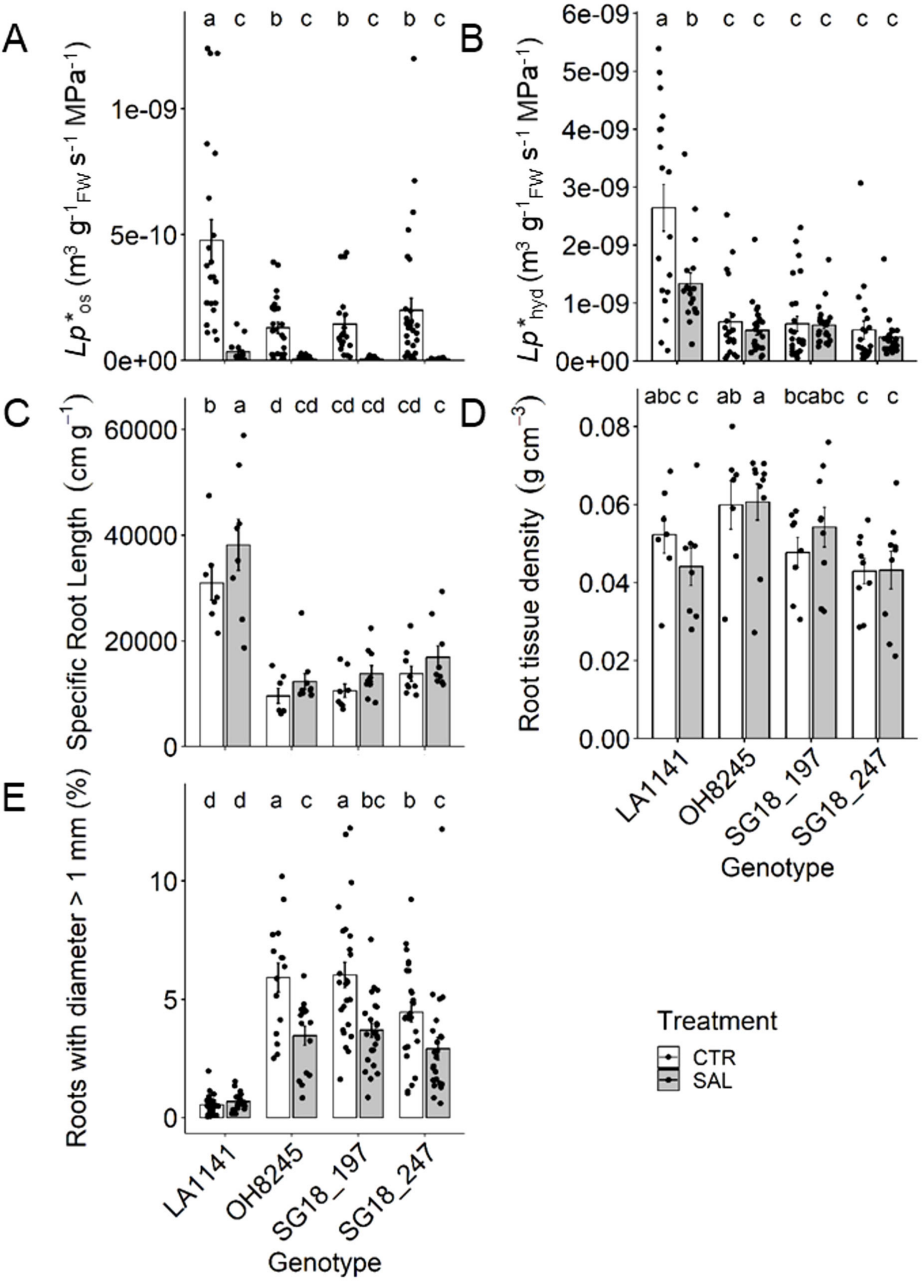
Root osmotic (Lp*_os_; **(A)**) and hydrostatic (Lp*_hyd_; **(B)**) hydraulic conductivity, specific root length **(C)**, root tissue density **(D)** and percent of roots with diameter >1 mm **(E)** after three to four weeks of control (1.5 dS m^-1^, CTR) and salinity treatment (12 dS m^-1^, SAL) of the tomato wild relative (*Solanum galapagense*; LA1141), tomato OH8245, and two introgression lines derived from their crossing (SG18_197 and SG18_247). Data show mean ± standard error (n =6-20). Different letters indicate statistical significance (α =0.05) based on linear mixed effect model followed by unrestricted LSD.

In general, LA1141 showed >70% higher root hydraulic conductivity (Lp*_r_) than the other genotypes, while OH8245 and the ILs had similar root Lp*_r_ (**Figure 1**). The salinity treatment decreased root osmotic hydraulic conductivity (Lp*_os_) by >93% in all genotypes, which is associated with cell-to-cell water transport driven by an osmotic gradient. The root hydrostatic hydraulic conductivity (Lp*_hyd_) decreased only in the wild relative LA1141 (49%), but it was still >69% higher than the other genotypes; Lp*_hyd_ measures the water movement driven by water tension in the soil-plant-atmosphere continuum (e.g., transpiration stream) (**Figures 1A, B**). LA1141 had >68% higher specific root length (SRL) than other genotypes, indicating longer roots (and higher area) per unit of root biomass. Overall, SRL increased under salinity (*p* value =0.02), but only LA1141-SAL had a significant 23% increase compared to its control (**Figure 1C**). Although root tissue density (RTD) was highly variable, OH8245 generally had 10% higher RTD than the other genotypes (*p* value =0.01) (**Figure 1D**), indicating higher root biomass per root length. LA1141 had the highest proportion of thin roots (i.e., <1 mm), and it maintained this proportion regardless of salinity treatment. Under salinity, OH8245 and the two ILs decreased the proportion of roots >1 mm by 35% (**Figure 1E**).

### Leaf gas exchange and plant water status

**Figure 2:**
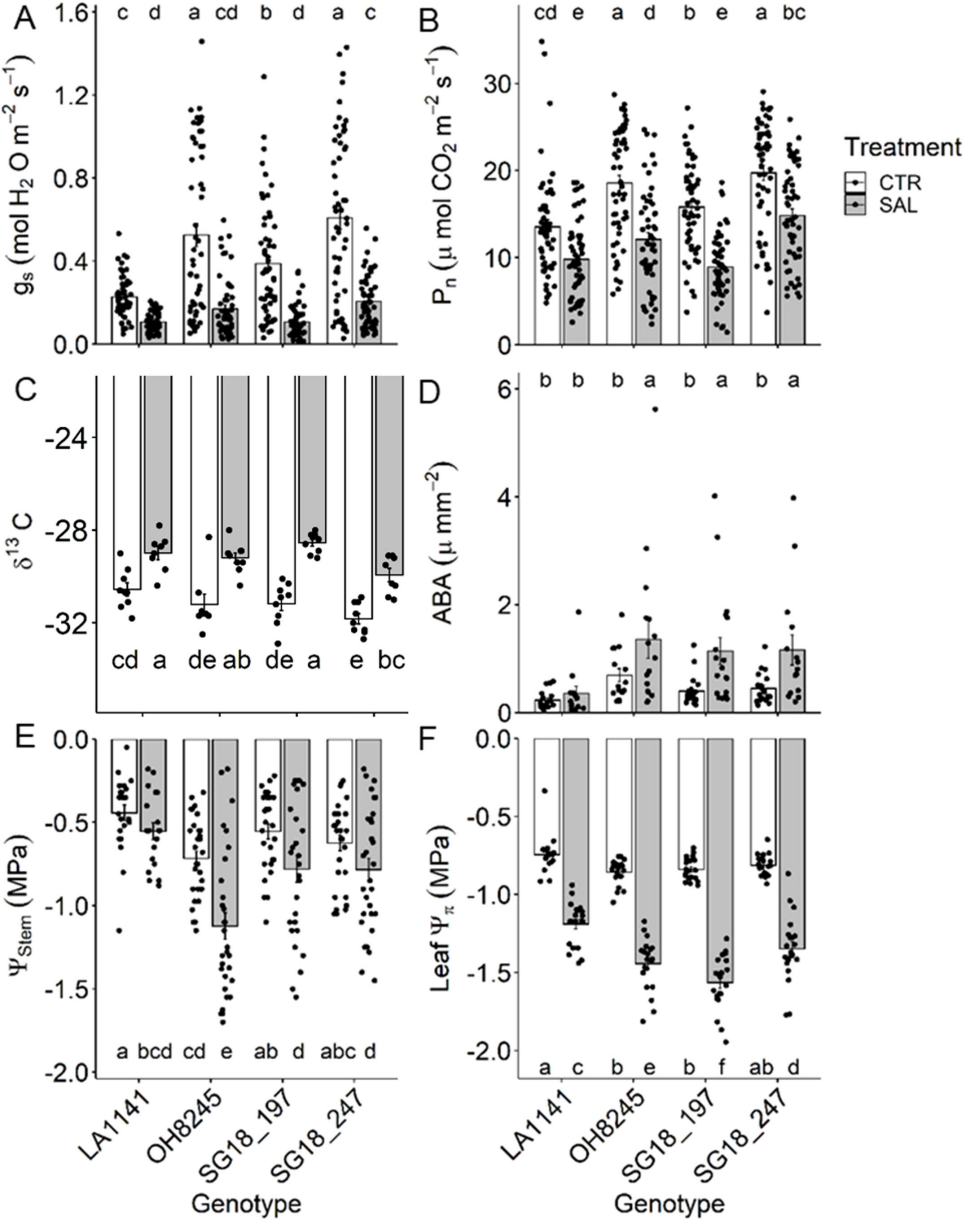
Stomatal conductance (g_s_; **(A)**), photosynthetic rate (P_n_; **(B)**), leaf δ^13^C **(C)**, leaf abscisic acid concentration **(D)**, stem water potential (Ψ_stem_; **(E)** and leaf osmotic potential (Ψπ; **(F)** after two to three weeks of control (1.5 dS m^-1^, CTR) and salinity treatment (12 dS m^-1^, SAL) of the tomato wild relative (*Solanum galapagense*; LA1141), tomato OH8245, and two introgression lines derived from their crossing (SG18_197 and SG18_247). Data show mean ± standard error (n =6-24). Different letters indicate statistical significance (α =0.05) based on linear mixed effect model followed by unrestricted LSD.

The salinity treatment decreased g_s_ (53% to 72%) more than P_n_ (23% to 43%) in all genotypes, resulting in >60% increase in intrinsic water use efficiency (**Figures 2A, B**). The stomatal conductance (g_s_) in LA1141 was half of OH8245 under control conditions, but similar under salinity because g_s_ decreased less in the wild-type tomato compared to OH8245. On the other hand, photosynthetic rate (P_n_) decreased by 23% in both LA1141 and OH8245, but the latter maintained higher P_n_ regardless of treatment. Under salinity, OH8245 had 26% higher P_n_ than SG18_197, and 23% lower P_n_ than SG18_247. The leaf *δ* ^13^C was 5% to 8% higher under salt stress, with SG18_247 having one of the more negative *δ* ^13^C values within treatments, which corroborates the higher g_s_ under salinity (**Figure 2C**). Night respiration increased in both ILs under salinity stress (44% to 51%), but it remained unchanged in LA1141 and OH8245 (**Supplementary Figure S3A**). LA1141 had 33% to 48% lower night respiration than OH8245, regardless of treatment. Overall, night transpiration decreased under salinity (*p* value =0.03), but only OH8245-SAL had a significant 68% decrease compared to its control (**Supplementary Figure S3B**). The percentage of C used for night respiration over the net C assimilation increased under salinity in OH8245 (three-fold) and SG18_197 (five-fold), but it remained stable in LA1141 and SG18_247 (**Supplementary Figure S3C**). Whereas leaf [ABA] was similar in LA1141 under both treatments, OH8245 and the ILs had two-fold increase in leaf [ABA] under salinity (**Figure 2D**).

The stem water potential (Ψ_stem_) was at least 29% higher in LA1141 and SG18_197 than OH8245, regardless of treatment (**Figure 2E**). SG18_247 was also able to maintain 43% higher Ψ_stem_ than OH8245 under salt treatment although both genotypes had similar Ψ_stem_ under the control treatment. The salinity treatment caused a >60% decrease in leaf Ψ*π* in all genotypes (**Figure 2F**). Under salinity, LA1141 had the highest (less negative) leaf osmotic potential (Ψ*π*), which was 24% higher than the lowest Ψ*π* observed in SG18_197. The salinity treatment decreased the stomatal density in all genotypes (36% to 38%), and LA1141 had consistently lower stomatal density than the other genotypes regardless of the salinity treatment (31% to 34%) (**Supplementary Figure S4A**). The salinity treatment decreased stomatal length (7% to 12%) and guard cell width (7% to 10%) in all genotypes but LA1141, which showed 10% higher stomatal size under salinity than other genotypes (**Supplementary Figures S4B, C**).

### Ion balance

**Figure 3:**
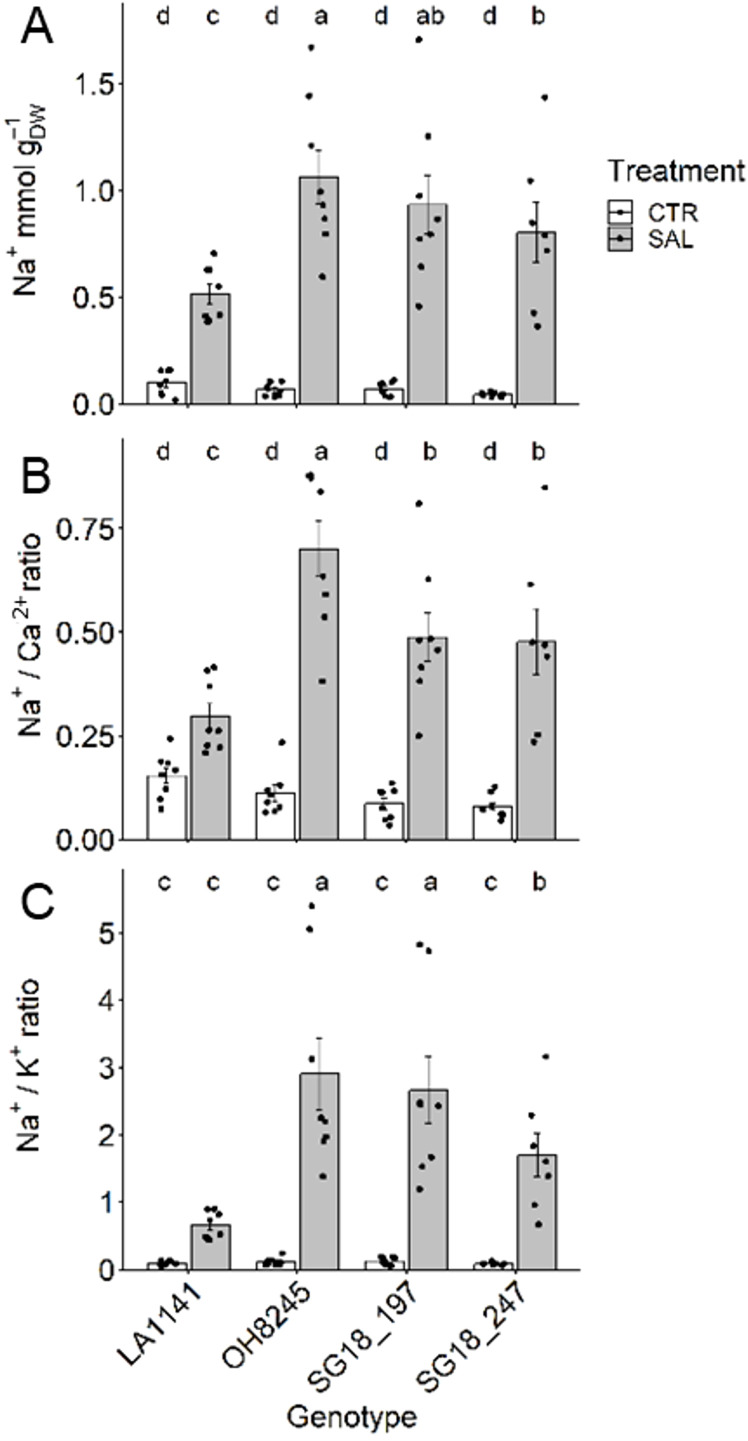
Leaf Na^+^ concentration **(A)**, Na^+^/Ca^2+^ ratio **(B)**, and Na^+^/K^+^ ratio **(C)** after two to three weeks of control (1.5 dS m^-1^, CTR) and salinity treatment (12 dS m^-1^, SAL) of the tomato wild relative (*S. galapagense*; LA1141), tomato OH8245, and two introgression lines derived from their crossing (SG18_197 and SG18_247). Data show mean ± standard error (n =6-8). Different letters indicate statistical significance (α =0.05) based on linear mixed effect model followed by unrestricted LSD.

Higher leaf [Na^+^] was found in all genotypes treated with salt (four to 18-fold increase), while no differences were observed under control conditions (average of 0.07 mmol g_DW_^-1^) (**Figure 3A**). Under salinity, OH8245 showed the highest leaf [Na^+^] (1.06 mmol g_DW_^-1^), whereas LA1141 had the least leaf [Na^+^] (0.51 mmol g_DW_^-1^). The SG18_247 was able to maintain 24% lower [Na^+^] than OH8245, but it was still 34% higher than LA1141. Under salt, Na^+^/Ca^2+^ ratio increased the most in OH8245 (five-fold higher) and the least in LA1141 (less than one-fold higher), while SG18_197 and SG18_247 had a Na^+^/Ca^2+^ ratio in between the parent lines (**Figure 3B**). A similar pattern was observed for Na^+^/K^+^ ratio, which increased 24-fold in OH8245 and only five-fold for LA1141 under salt. The ILs had on average 19-fold higher Na^+^/K^+^ ratio, with SG18_197 having a similar Na^+^/K^+^ ratio than OH8245, and SG18_247 in between the parent lines (**Figure 3C**). Leaf nutrient profile changed among genotypes and treatments. Leaf N was 30% to 47% higher in LA1141 compared to the other genotypes, and it decreased 12% due to salinity only in LA1141. Leaf N increased 20% and 50% under salinity in OH8245 and SG18_247, respectively (**Supplementary Figure S2D**). The IL SG18_247 showed upregulation of two high-affinity nitrate transporters (*Solyc06g010250* and *Solyc11g069750*, **Supplementary Table S3**), which could partly explain this high increase in N under salinity. LA1141 had higher Cu (47% to 50%) and K^+^ (42% to 50%) than OH8245 regardless of the treatment (**Supplementary Figure S5**). Under salinity treatment, Ca^2+^ increased >1.4-fold in all genotypes, Cu increased (>57%) in all genotypes except for SG18_197, while K^+^ decreased (>18%) in all genotypes except for SG18_247, and Mg^2+^ decreased (>57%) in all genotypes (**Supplementary Figure S5**).

### Biomass

**Figure 4:**
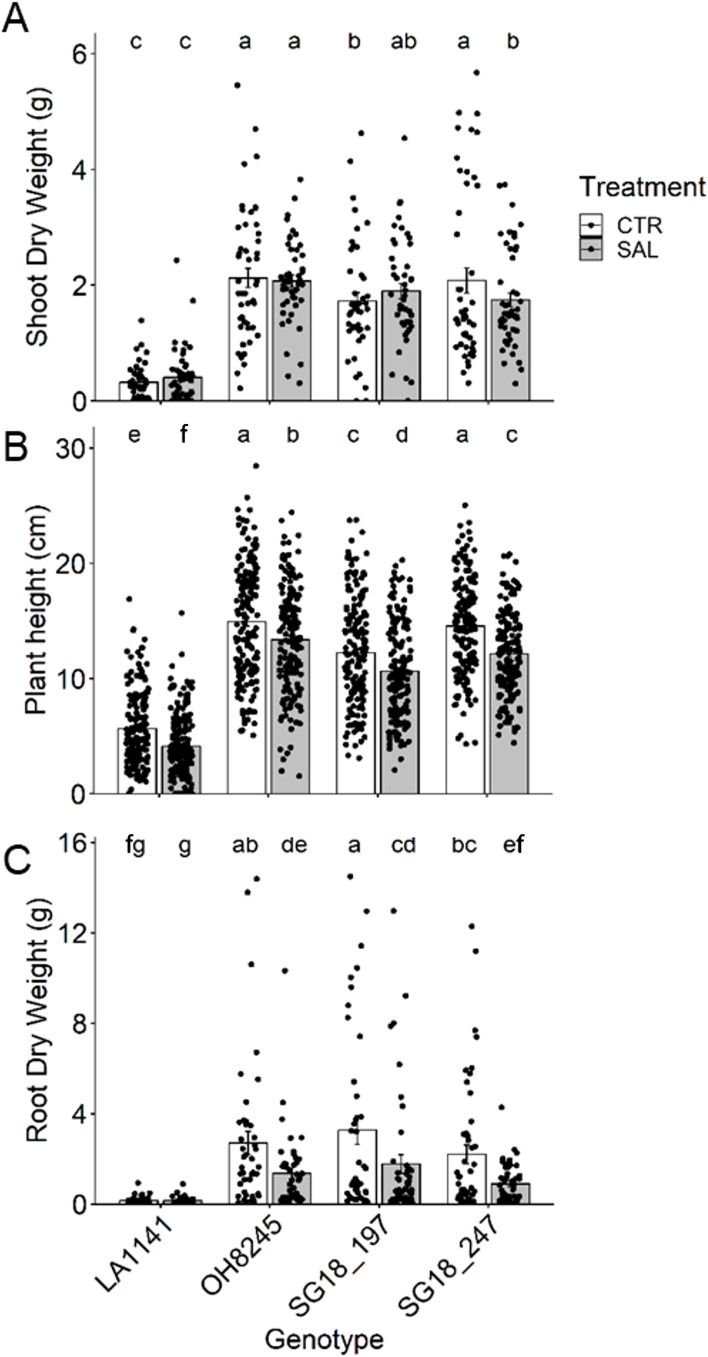
Shoot dry weight **(A)** plant height **(B)**, and root dry weight **(C)** after three to four weeks of control (1.5 dS m^-1^, CTR) and salinity treatment (12 dS m^-1^, SAL) of the tomato wild relative (*Solanum galapagense*; LA1141), tomato OH8245, and two introgression lines derived from their crossing (SG18_197 and SG18_247). Data show mean ± standard error (n =48). Different letters indicate statistical significance (α =0.05) based on linear mixed effect model followed by unrestricted LSD.

The three-week salinity treatments were successful in affecting plant physiological performance (and molecular expression) without imparting a drastic response to extreme stress (e.g., plant toxicity and death). Shoot dry weight (DW) was not affected except in IL SG18_247 (16% decrease; **Figure 4A**), but plant height decreased 11% to 27% in all genotypes (**Figure 4B**). Root DW decreased 46% to 59% in all genotypes except for the wild relative LA1141 (**Figure 4C**). Overall, LA1141 showed five to 16 times lower root and shoot DW than any of the other three genotypes, regardless of treatment. The relative growth rate (RGR) of LA1141 was less than half of any other genotype, regardless of the treatment (**Supplementary Figure S2A**). OH8245 had ~15% higher RGR than both ILs, but under salinity, SG18_197 increased RGR and was similar to OH8245. SLA was not affected by the salinity treatment (**Supplementary Figure S2B**). Generally, LA1141 had >50% higher SLA than OH8245, and SG18_197 had ~17% higher SLA than OH8245. LA1141 had >30% higher leaf [N] than the other genotypes, which is usually correlated with higher SLA across plant species (Lambers et al., 2008) (**Supplementary Figure S2D**).

### RNA-seq

**Figure 5:**
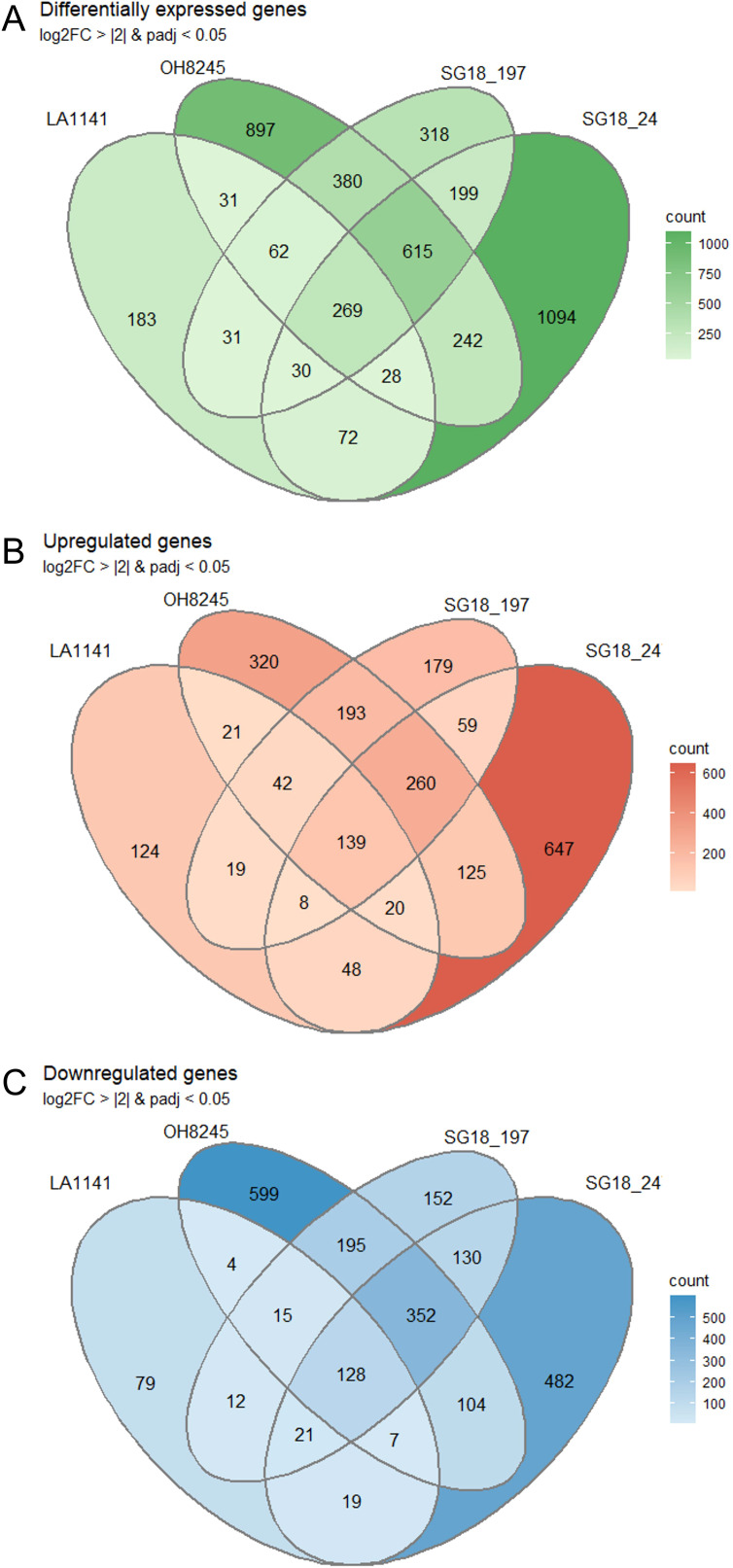
The Venn diagrams represent the overlap between sets of differentially expressed genes (DEGs) from LA1141, OH8245, SG18_197 and SG18_247 in response to three weeks of salinity treatment (12 dS m^-1^) compared to their controls (1.5 dS m^-1^). Shown are all DEGs **(A)**, upregulated genes **(B)**, and downregulated genes **(C)**. DEGs were defined by adjusted *p* value <0.05 and |log_2_FC| >2.

A total of approximately 1.4 billion raw reads were generated utilizing the Illumina high-throughput platform. After trimming for adapters and quality, the 24 libraries had between 46.9 and 63.3 million sequences each. 75.8% to 85.3% of the reads were successfully assigned to SL4.0 transcripts (**Supplementary Table S4**). The heat map identified clusters of genes that show coordinated behavior (**Supplementary Figure S6**). Each genotype reacted notably different to the salt stress as evident by the varying numbers of DEGs identified in each strain. (**Figure 5A**). The wild relative LA1141 had the lowest number of DEGs between treatments (706), followed by SG18_197 (1904), OH8245 (2524) and SG18_247 (2549). Under salt, the wild relative LA1141 had more upregulated genes (421) (**Figure 5B**) than downregulated genes (285) (**Figure 5C**), but all other genotypes had more downregulated genes (1005 for SG18_197, 1321 for SG18_247, and 1404 for OH8245) than upregulated genes (899 for SG18_197, 1306 for SG18_247, and 1120 for OH8245) compared to their control treatment (**Figure 5**). The high correlation (R^2^ = 0.93; *p* value <0.0001) (**Supplementary Figure S7**) between RT-PCR and RNA-seq results supports the reliability of the RNA-Seq analysis (Luo et al., 2017).

In order of significance, the enriched molecular functions were transmembrane transporter activity (GO:0022857), transporter activity (GO:0005215), and inorganic molecular entity transmembrane transporter activity (GO:0015318) for LA1141, and catalytic activity (GO:0003824), oxidoreductase activity (GO:0016491), and transmembrane transporter activity (GO: 0022857) for the other genotypes (**Table 1**). The enriched biological processes and cellular components are listed in **Supplementary Tables S5**, **S6**, respectively. While the LA1141 showed some molecular functions that were not enriched in the ILs (e.g., GO:0015318, GO:0015075, GO: 1901702, which play an important role in ion balance under salinity), the ILs showed unique enriched molecular functions and biological processes, including lyase activity (GO:0016829) for the IL SG18_197 and nitrate and amino acid transporter activity for SG18_247 (GO:0015112 and GO:0015171) as molecular functions and inorganic anion transmembrane transport and lipid storage (GO:0098661 and GO:0019915) for SG18_247 in biological processes.

**Table 1:** Enriched molecular functions of differentially expressed genes between control and salinity treatment in each genotype, and respective *p* values when <0.05 (white represents *p* values closer to 0.05 and red further from 0.05) (*p* values are adjusted using Bonferroni method).

	LA1141		OH8245		SG18 197		SG18 247	
	Fold enrichment	*p* value	Fold enrichment	*p* value	Fold enrichment	*p* value	Fold enrichment	*p* value
transmembrane transporter activity (GO:0022857)	3.09	1.26E-05	2.08	4.15E-06	2.37	3.91E-07	2.41	1.29E-10
transporter activity (GO:0005215)	2.96	3.25E-05	2.02	1.22E-05	2.31	8.96E-07	2.31	1.25E-09
inorganic molecular entity transmembrane transporter activity (GO:0015318)	4.43	6.79E-04						
molecular_function (GO:0003674)	1.38	1.97E-03	1.40	1.00E-15	1.39	3.14E-11	1.34	2.68E-11
monoatomic ion transmembrane transporter activity (GO:0015075)	3.69	3.75E-02						
salt transmembrane transporter activity (GO: 1901702)	4.66	4.10E-02						
glycosyltransferase activity (GO:0016757)	3.62	4.57E-02	2.43	1.33E-02			2.39	1.49E-02
monoatomic cation transmembrane transporter activity (GO:0008324)	3.85	4.80E-02						
catalytic activity (GO:0003824)			1.43	1.74E-08	1.49	4.82E-08	1.50	1.18E-11
oxidoreductase activity (GO:0016491)			2.04	2.14E-06	2.07	6.94E-05	2.43	3.00E-12
UDP-glucosyltransferase activity (GO 0035251)			4.71	1.93E-04			4.91	5.58E-05
glucosyltransferase activity (GO:0046527)			4.04	2.00E-04			3.61	3.01E-03
molecular function regulator activity (GO:0098772)			2.44	6.18E-04				
enzyme inhibitor activity (GO:0004857)			3.55	7.45E-04			3.34	2.92E-03
molecular function inhibitor activity (GO:0140678)			3.55	7.45E-04			3.34	2.92E-03
structural molecule activity (GO:0005198)			0.06	8.35E-04			0.06	8.50E-04
R binding (GO:0003723)			0.33	1.18E-03	0.21	2.12E-04	0.25	2.15E-05
UDP-glycosyltransferase activity (GO:0008194)			3.70	1.24E-03			4.01	1.20E-04
hormone binding (GO:0042562)			7.86	1.81E-03	8.46	7.95E-03	0.30	4.88E-05
hexosyltransferase activity (GO:0016758)			2.84	2.27E-03			2.60	2.85E-02
signaling receptor activity (GO:0038023)			4.80	2.99E-03	6.00	5.78E-04	6.77	2.84E-07
molecular transducer activity (GO:0060089)			4.42	3.36E-03	5.16	2.47E-03	5.81	2.44E-06
enzyme regulator activity (GO:0030234)			2.37	3.58E-03				
sequence-specific D binding (GO:0043565)			1.84	6.51E-03	2.24	6.56E-05		
protein phosphatase inhibitor activity (GO:0004864)			6.50	6.95E-03	7.00	2.46E-02	7.70	2.43E-04
phosphatase inhibitor activity (GO:0019212)			6.29	8.85E-03	6.77	3.02E-02	7.44	3.24E-04
carboxylic ester hydrolase activity (GO:0052689)			3.09	1.26E-02				
Iyase activity (GO:0016829)					3.15	1.28E-02		
transferase activity, transferring alkyl or aryl (other than methyl) groups (GO:0016765)					3.09	1.62E-02		
D -binding transcription factor activity (GO:0003700)					1.97	2.46E-02		
organic acid binding (GO:0043177)					5.86	2.83E-02	6.20	6.09E-04
carboxylic acid binding (GO:0031406)					5.86	2.83E-02	6.20	6.09E-04
monooxyge se activity (GO:0004497)							3.69	7.52E-04
acyltransferase activity, transferring groups other than amino-acyl groups (GO:0016747)							2.60	3.25E-03
structural constituent of ribosome (GO:0003735)							0.07	3.88E-03
hydrolase activity, acting on ester bonds (GO:0016788)							1.88	5.38E-03
carboxylic acid transmembrane transporter activity (GO:0046943)							3.92	6.41E-03
organic acid transmembrane transporter activity (GO:0005342)							3.92	6.41E-03
protein phosphatase regulator activity (GO:0019888)							4.85	1.23E-02
phosphatase regulator activity (GO:0019208)							4.75	1.47E-02
protein binding (GO:0005515)							0.39	1.57E-02
organic anion transmembrane transporter activity (GO:0008514)							3.40	1.72E-02
nitrate transmembrane transporter activity (GO:0015112)							11.16	3.67E-02
amino acid transmembrane transporter activity (GO:0015171)							3.84	4.68E-02

### Candidate salt tolerance-related genes

The salt tolerance response mechanisms displayed by LA1141, and identified in this study, were reflected by 40 genes uniquely DE in LA1141 and fewer DEGs in LA1141 compared to OH8245 (**Table 2**). Within these genes, nine were DE in SG18_197, including one apyrase (*Solyc02g032550*) involved in cellular ATP homeostasis (Liu et al., 2019), two MADS-box transcription factors (TFs) (*Solyc02g091550* and *Solyc01g087990*), a GRAS TF (*Solyc09g018460*), a NF-Y TF (*Solyc07g065500*), and one Na^+^ transporter (*Solyc07g014690*). Within the 40 uniquely DEGs in LA1141, sixteen genes were DE in SG18_247, including one Casparian strip membrane protein (*Solyc10g083250*), two phosphate transporters (*Solyc08g007800*, *Solyc06g072510*), a nitrate transporter (*Solyc08g007430*), the SlDREB1 (*Solyc06g050520*), a *SlCRF1/PTI6* gene (*Solyc06g082590*), a *NF-Y TF* (*Solyc07g065500*), a poly (ADB-ribose) polymerase (*Solyc01g009470*), a plant invertase/pectin methylesterase inhibitor superfamily protein (*Solyc08g079235*), a phospholipase D (*Solyc08g066790*), a type I inositol polyphosphate 5-phosphatase (*Solyc01g005090*), and a MAP kinase (*Solyc02g090430*), all involved in drought and salinity stress responses (Ogawa et al., 2009; Coculo and Lionetti, 2022; Hong et al., 2010; Kaye et al., 2011; Zhang et al., 2014).

**Table 2:** List of genes involved in enriched biological processes under salinity, and were differentially expressed in LA1141, but not in OH8245. Log_2_FC =NA indicates that adjusted *p* value (p-adj) >0.05.

		LA1141		SG18_197		SG18_247	
Gene ID	Description	log_2_FC	p-adj	log_2_FC	p-adj	log_2_FC	p-adj
*Solyc01g005090*	Type I inositol polyphosphate 5-phosphatase 5	-1.6	0.0	NA	0.8	-2.7	0.0
*Solyc01g009470*	Poly [ADP-ribose] polymerase	4.4	0.0	NA	0.7	5.2	0.0
*Solyc01g087990*	MADS-box transcription factor	2.1	0.0	1.7	0.0	NA	0.2
*Solyc01g097850*	Agamous-like MADS-box protein AGL.1	3.4	0.0	NA	0.8	NA	0.4
*Solyc02g032550*	Apyrase	2.1	0.0	1.5	0.0	NA	0.3
*Solyc02g036370*	Protein REVEILLE 7-like	-2.9	0.0	NA	0.1	NA	0.6
*Solyc02g080540*	ATP synthase gamma chain, chloroplastic	2.0	0.0	NA	0.3	NA	0.4
*Solyc02g090430*	MAP kinase kinase kinase 20	2.2	0.0	NA	0.4	-2.7	0.0
*Solyc02g091550*	MADS box transcription factor AGAMOUS	1.6	0.0	2.9	0.0	NA	0.6
*Solyc03g113930*	22.0 kDa class IV heat shock protein	4.6	0.0	NA	0.3	NA	0.8
*Solyc04g063350*	2-oxoisovalerate dehydrogenase subunit alpha 2, mitochondrial	5.3	0.0	NA	0.6	NA	0.5
*Solyc05g013510*	Phosphate transporter	3.5	0.0	NA	0.2	NA	0.5
*Solyc05g056050*	Chlorophyll a-b binding protein	2.7	0.0	NA	1.0	NA	0.2
*Solyc06g050520*	DREB protein 1	3.8	0.0	NA	0.4	3.4	0.0
*Solyc06g062430*	Inositol oxygenase	-2.9	0.0	NA	0.6	NA	0.8
*Solyc06g072510*	Phosphate carrier protein, mitochondrial	-3.1	0.0	NA	0.2	-2.9	0.0
*Solyc06g082590*	DNA-binding protein Pti6	-1.5	0.0	NA	0.3	-2.2	0.0
*Solyc07g014690*	Na^+^ transporter HKT1;1	5.8	0.0	3.1	0.0	NA	0.7
*Solyc07g053360*	Late embryogenesis abundant protein	-2.6	0.0	NA	0.6	NA	0.1
*Solyc07g065500*	Nuclear transcription factor Y subunit B-3	2.5	0.0	3.5	0.0	3.2	0.0
*Solyc08g007430*	NIT2	-2.9	0.0	NA	0.8	2.8	0.0
*Solyc08g007800*	SPX domain-containing protein	-4.8	0.0	NA	0.2	-6.3	0.0
*Solyc08g066790*	Phospholipase D	-4.1	0.0	NA	0.6	0.4	0.0
*Solyc08g079235*	Plant invertase/pectin methylesterase inhibitor superfamily protein	2.1	0.0	NA	0.8	2.5	0.0
*Solyc09g018460*	GRAS5	2.2	0.0	1.8	0.0	NA	0.5
*Solyc10g083250*	Casparian strip membrane protein.1	1.6	0.0	NA	0.5	3.0	0.0
*Solyc12g042830*	Class I heat shock protein	4.2	0.0	NA	0.5	NA	0.2

### Transcriptome SNP analysis

**Figure 6:**
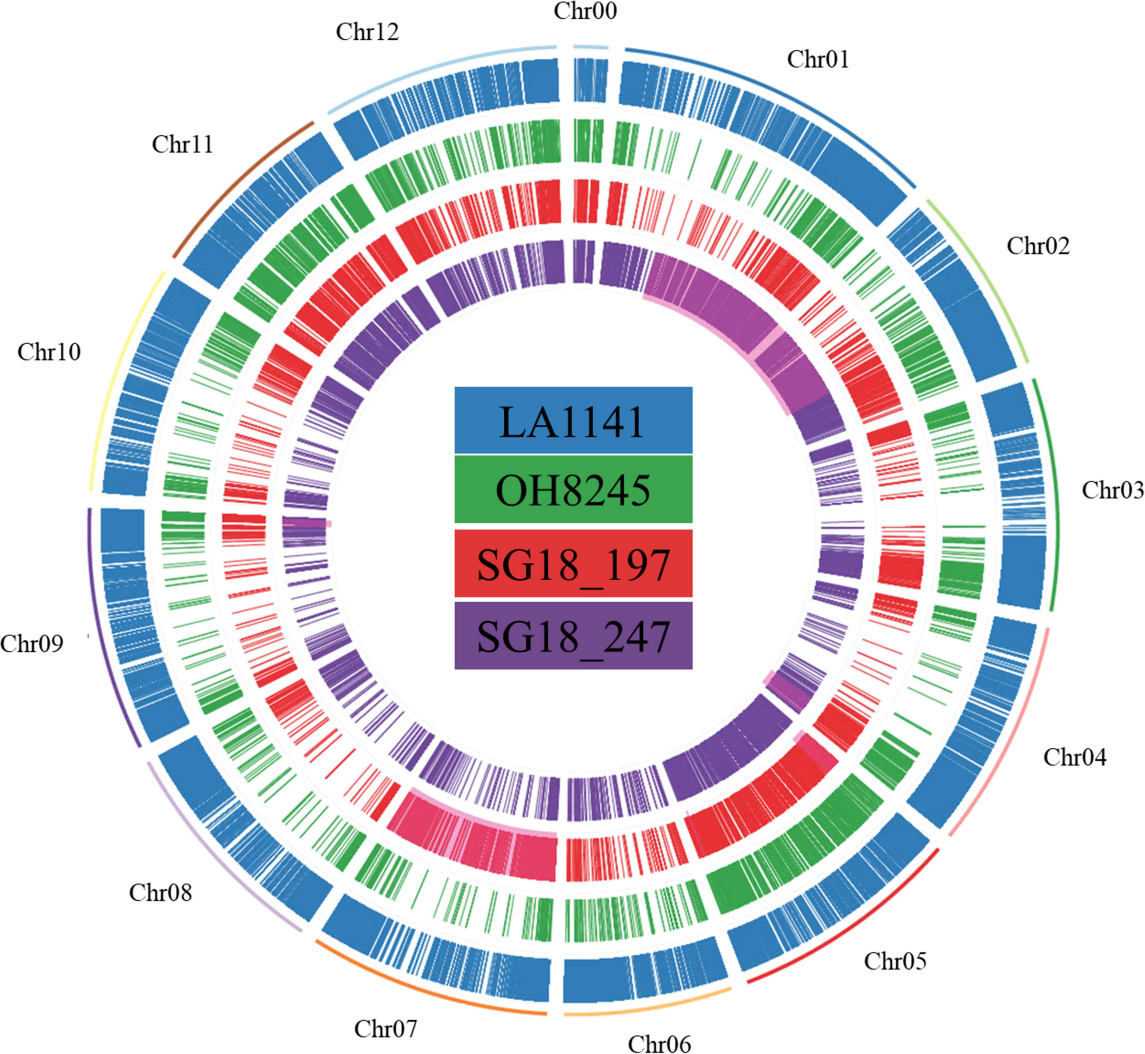
The circular diagram represents the transcriptome SNPs in LA1141, OH8245, SG18_197 and SG18_247 compared to the tomato reference genome Sl4.0. SNPs were considered only if homozygous and Phred score was ≥25. The portion of the genome of the introgression lines highlighted in red represents the introgression segments based on their genetic map.

A total of 112575, 12808, 21337, and 24904 transcriptome SNPs were found for LA1141, OH8245, SG18_197 and SG18_247, respectively. A higher SNP density was observed at the distal portion of each chromosome. The highest SNP density for LA1141 was found in chromosome 2, followed by chromosome 6 and 3. For OH8245, the highest SNP density was found in chromosome 5, followed by chromosome 11 and 4 (**Supplementary Figure S8**). As expected, the ILs had higher SNPs in the respective introgression segments (chromosome 5 and 7 for SG18_197 and 1 and 2 for SG18_247) (Fenstemaker et al., 2022b) (**Figure 6**). Only 452 to 521 SNPs were classified as high impact variants by the SnpEff analysis in each genotype (**Supplementary Table S7**). The LA1141 and the ILs SG18_197 and SG18_247 had 19 and 25 high-impact SNP in common (**Supplementary Table S8**). To note are the RING/FYVE/PHD-type zinc finger family gene (*Solyc01g087400*), PTEN2A-like gene (*Solyc01g107750*), Golgin candidate 6 (*Solyc02g084490*) involved in ion homeostasis and salinity response in SG18_197, and the chlorophyll-related protoporphyrinogen oxidase (*Solyc01g079090*), metal transporter Nramp3 (*Solyc03g116900*), and UDP-galactose/UDP-glucose transporter 2-like (*Solyc08g080270*) in SG18_247.

## Discussion

This study shows how the introgression lines improved their salinity tolerance partly due to better ion balance, leaf gas exchange, plant water status and/or root morphology than the cultivated tomato used as a parent. The salinity treatment used in this study allowed us to detect long-term responses to salinity stress under conditions that prevent an immediate toxic effect of high Na^+^ concentration, which is not common under production systems (Grattan and Grieve, 1998). The physiological characterization showed that the ILs differed in their response mechanisms and tradeoffs in performance, which highlights that multiple strategies may be possible and necessary to achieve salinity tolerance within a crop species. The root transcriptome allowed us to identify potential gene candidates and SNPs to use in tomato breeding programs aiming at improving plant water relations and ion balance under salinity, and warrants further investigation for this set of genes.

The wild relative LA1141 mainly displayed an exclusion response to salinity, driven by root characteristics such as higher SRL (e.g., higher root length per unit of dry weight) that increased root surface area and conferred better water status and nutrient uptake capacity, and root ion transporters which supported Na^+^ exclusion and higher Ca^2+^ and K^+^ leaf content (**Supplementary Table S3**). In addition, the low number of DEGs, low stomatal density and the unchanged leaf [ABA] of LA1141 supports a seemingly constitutive response to salinity stress tolerance in LA1141, but the tradeoff being low plant biomass. Yet, increasing salinity tolerance in tomatoes should be accomplished with little or no impact on plant productivity. The IL SG18_197, whose introgression region is on chromosome 7, also had a low number of DEGs compared to the recurrent parent OH8245, indicating that chromosome 7 harbors important molecular traits involved in the tomato response to prolonged salinity stress (e.g., *HKT1;1* or *Solyc07g014690*; Asins et al., 2013).

The tomato variety OH8245 was unable to regulate Na^+^ homeostasis and had higher number of DEGs than LA1141 and SG18_197. The transcriptome showed that even after 21 DOT, OH8245 was still actively responding to stress, as supported by the high number of differentially expressed heat shock protein genes (Wang et al., 2004). Under an entire production season, we speculate that OH8245 may not be able to cope with a persistent salinity stress as [Na^+^] impairs physiological activity (Munns and Tester, 2008). On the other hand, SG18_247, whose introgression region is on chromosome 1 and 2, showed a high number of DEGs, but its response was enriched in phosphate and nitrate transporters, amino acid catabolism, Casparian strip membrane proteins, plant invertases and pectin methylesterases, which supported C assimilation with low C maintenance costs (**Supplementary Figure S3C**). Slight decreases in shoot biomass accompanied with higher P_n_ and better ion homeostasis in SG18_247 account for inherent but minimal tradeoffs to a multi-trait salinity response.

### Plant water relations and leaf gas exchange

One plant mechanism to cope with salinity is increasing the root surface area by decreasing root diameter (Huang and Eissenstat, 2000). This was consistent in all genotypes but LA1141, which already had thinner roots, higher Lp*_r_ and higher SRL under control conditions. On the other hand, OH8245 maintained a high RTD and decreased root diameter, indicating suberin and lignin deposition in roots and an overall lower Lp*_hyd_ (Hernandez-Espinoza and Barrios-Masias, 2020). At the molecular level, the contribution of different genes expressing aquaporin, peroxidase, Casparian strip membrane protein, laccase and late embryogenesis abundant proteins contributed to higher Lp*_r_ of LA1141 under salinity (**Supplementary Table S3**). SG18_247 had some similarities to LA1141, such as the upregulation of several Casparian strip membrane proteins, lower RTD and thinner roots, which can help maintain a better plant water status (Lovelli et al., 2012; Bonarota et al., 2024a).

A better capacity for root water uptake (e.g., higher *Lp*_r_ and SRL) can support shoot transpiration demands and C assimilation, especially when water loss driven by higher g_s_ can lower Ψ_stem_ and reduce growth rates. Overall, LA1141 maintained a better plant water status (e.g., higher Ψ_stem_), but had a lower leaf gas exchange capacity than the other genotypes, which may be partly due to a lower stomatal density. Other confounding factors affecting C assimilation capacity are costs associated with salinity tolerance such as resource allocation (e.g., N) to cell-wall hardening (Degenhardt and Gimmler, 2000; Bonarota et al., 2024b). Although both ILs maintained a similar Ψ_stem_ under salinity, SG18_247 maintained higher leaf gas exchange and C assimilation capacity, providing more C assimilates for maintenance and growth. Under salinity, the decrease in P_n_ can be affected by an ionic effect (Zhang et al., 2022), but SG18_247 maintained a higher P_n_ likely supported by a lower leaf Na^+^/K^+^ ratio (Shabala and Cuin, 2008; Rawat et al., 2022). Studies on tomato genotypes with higher P_n_ under salinity are lacking, and SG18_247 can be a good genetic resource for the identification of genes and physiological mechanisms of gas exchange in salinity tolerance breeding (Nebauer et al., 2013; Dariva et al., 2020).

Mitochondrial respiration for the maintenance of the membrane potential gradient and metabolic activities (e.g., osmotic adjustment) could consume up to 60% of the assimilated C (Munns et al., 2020; Poorter et al., 1990; Jacoby et al., 2011). One mitochondrial phosphate carrier (*Solyc06g072510*), likely involved in ATP production (Paul et al., 2016) was downregulated in SG18_247 and LA1141 and could play a role in tomato salinity tolerance. The wild relative *S. chilense* increases the expression of genes involved in both photosynthetic and respiratory rates to compensate for the higher C demand under salinity (Zhou et al., 2011). In this study, respiration rates were maintained in the parent lines, but respiration increased for the ILs under salinity, suggesting an active response and increased C costs (**Supplementary Figure S3C**). In the long term, this higher C cost could reduce growth rates and crop performance at the fruit filling stage (e.g., melons; Lima et al., 2020), but further studies are needed.

### Nutrient balance

Salinity stress alters plant nutrient uptake, with consequent nutrient deficiencies (e.g., NO_3_^-^ and K^+^; Grattan and Grieve, 1998) and ion toxicities (e.g., Na^+^ and Cl^-^; Munns et al., 2016). The wild relative LA1141 had lower Na^+^, higher N, K^+^, Ca^2+^ and Cu than OH8245 under salinity, displaying a strategy to maintain ion homeostasis through regulation of transporters at the root level (**Supplementary Table S3**). It is known that lower leaf Na^+^ content improves photosynthetic performance (Montesano and van Iersel, 2007), and it may be partly a reason for the higher P_n_ of SG18_247 under salinity. In addition, SG18_247 maintained [K^+^] similar to control conditions, which supports stomatal regulation and enzyme activity (Marschner, 1974; Tahal et al., 2000). *Solyc01g098190*, a Na^+^/K^+^ exchanger (**Supplementary Table S3**), was upregulated in all genotypes, and it could play a role in the Na^+^/K^+^ homeostasis and increase salinity tolerance. Leaf N content is positively correlated with chlorophyll content and photosynthetic efficiency (Sun et al., 2022), but LA1141 did not improve P_n_ even when leaf total N was ~40% higher than the other genotypes. This may be due to N allocation to structural components in the cell wall or N being stored in inorganic form (Bonarota et al., 2024b; Degenhardt and Gimmler, 2000). The upregulation of a high-affinity nitrate transporter under salinity, *Solyc11g069750*, could explain the increase in leaf N in LA1141 and SG18_247. A field trial using the IL SG18_197 as a tomato rootstock showed its capacity to sustain higher leaf N content even under no N fertilization (Bonarota and Barrios-Masias, 2024), supporting the value of this genetic resource in low-input agricultural systems. Calcium can improve Na^+^/K^+^ ratio, maintain cell membrane integrity, and act as a messenger in signal transduction pathways under salinity (Cramer, 2002). In our study, a higher Na^+^/Ca^2+^ ratio in OH8245 may result in higher salinity toxicity when OH8245 is exposed to salinity during a full growing season. *Solyc07g006370*, a cation/Ca^2+^ exchanger, could explain the higher leaf [Ca^2+^] in LA1141 and SG18_197. The role of the plant nutrient profile and their regulation at the molecular level should be further studied as it is the case with Cu, which increased in all genotypes, and it is likely involved in reactive oxygen species scavenging (Hejazi et al., 2012). *Solyc08g061610*, a Cu-transporting ATPase PAA2 could explain the higher leaf [Cu] in LA1141 (**Supplementary Table S3**).

## Conclusion

This study provides physiological and molecular insights into tomato salinity response mechanisms such ion homeostasis, plant water relations, leaf gas exchange and root morphology. The tomato wild relative LA1141 showed high water and ion uptake capacity, root surface area, and low stomatal density, with the tradeoff of lower biomass. The introgression of LA1141 genomic regions into OH8245 in SG18_197 and SG18_247 resulted in a complex suite of salinity response mechanisms with better plant water status under salinity, improving salinity tolerance. In SG18_197, the introgression resulted in better Na^+^/Ca^2+^ ratio, and less root DEGs, with the tradeoff of lower photosynthetic rate and leaf osmotic potential (higher energy costs). In SG18_247, the introgression resulted in higher photosynthetic rate, lower Na^+^/K^+^ ratio, and transcriptomic rearrangements involving nitrate and phosphate transporters, and Casparian strip membrane proteins, with the tradeoff of a slight decrease in biomass accumulation than OH8245 under salinity. Each IL warrants further evaluation on their plasticity under an array of soil salinity conditions and could be tested in open field conditions. The traits and genes identified in this study could help breeding programs in improving ion balance and plant water relations under salinity stress in tomato, although further investigation is needed to confirm the gene role and impact in salinity tolerance.

## Data Availability

The datasets presented in this study can be found in online repositories. The names of the repository/repositories and accession number(s) can be found below: https://www.ncbi.nlm.nih.gov/, SAMN42375299 to SAMN42375322.
