# Supplementary material for: Physiology of salt tolerance introgressions from Solanum galapagense in the domesticated tomato
Source: Front Plant Sci. 2025 May 15;16:1568851. doi: 10.3389/fpls.2025.1568851 (PMC12119524; doi:10.3389/fpls.2025.1568851)
Supplement: Supplementary file 1 [file DataSheet1.docx]

**Figure S1.** Physiological screening of the *S. galapagense* LA1141, the processing tomato cultivar OH8245, and ten introgression lines under control (CTR) and 12 dS m^-1^ (SAL).


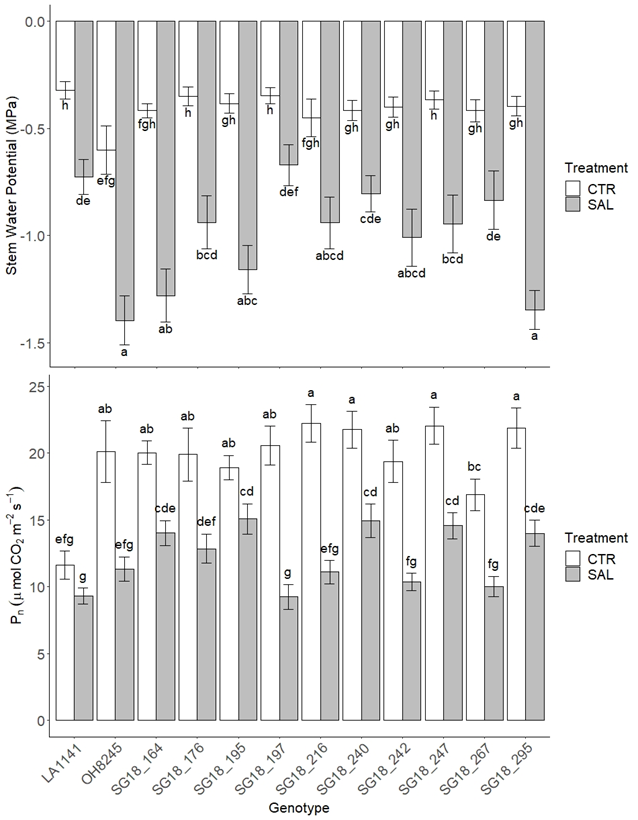


**Table S1**. Library size and quality of alignment to four different genomes of each sample.

|  |  | **Reference SL4.0** | | | ***S. lycopersicum* OH8245** | | | ***S. pimpinellifolium* LA2093** | | | ***S. pimpinellifolium* LA1670** | | |
| --- | --- | --- | --- | --- | --- | --- | --- | --- | --- | --- | --- | --- | --- |
| **Genotype** | **Treatment** | **% Proper Pairs** | **% Assigned** | **M Assigned** | **% Proper Pairs** | **% Assigned** | **M Assigned** | **% Proper Pairs** | **% Assigned** | **M Assigned** | **% Proper Pairs** | **% Assigned** | **M Assigned** |
| **LA1141** | **CTR** | 93.8% | 83.3% | 47.4 | 93.1% | 82.1% | 46.9 | 94.3% | 79.8% | 45.2 | 92.2% | 85.7% | 48.9 |
|  |  | 92.2% | 83.3% | 51.9 | 91.7% | 81.6% | 51.0 | 93.2% | 79.4% | 49.1 | 91.7% | 86.0% | 53.5 |
|  |  | 88.6% | 79.1% | 45.0 | 90.6% | 79.4% | 44.3 | 92.8% | 78.7% | 42.6 | 90.6% | 84.3% | 46.4 |
|  | **SAL** | 92.2% | 81.9% | 50.2 | 91.0% | 80.3% | 49.6 | 92.6% | 78.2% | 47.8 | 90.5% | 83.9% | 51.7 |
|  |  | 93.3% | 83.4% | 50.3 | 92.6% | 82.3% | 49.8 | 93.7% | 79.8% | 48.0 | 92.0% | 86.2% | 52.1 |
|  |  | 93.1% | 83.4% | 40.0 | 92.3% | 82.1% | 39.5 | 93.5% | 79.6% | 38.0 | 91.7% | 85.7% | 41.2 |
| **OH8245** | **CTR** | 84.1% | 75.8% | 40.9 | 85.0% | 74.8% | 39.9 | 89.6% | 76.3% | 38.9 | 87.6% | 82.1% | 42.0 |
|  |  | 90.4% | 81.7% | 42.6 | 90.3% | 80.6% | 42.0 | 91.9% | 78.7% | 40.3 | 90.3% | 85.0% | 43.8 |
|  |  | 87.7% | 79.2% | 41.6 | 88.0% | 78.4% | 41.0 | 90.2% | 76.8% | 39.2 | 88.4% | 82.9% | 42.7 |
|  | **SAL** | 95.5% | 85.2% | 49.2 | 95.2% | 84.6% | 49.0 | 95.6% | 81.6% | 47.0 | 93.7% | 87.5% | 50.7 |
|  |  | 95.2% | 85.2% | 52.6 | 94.6% | 84.4% | 52.3 | 95.3% | 81.4% | 50.0 | 93.2% | 87.2% | 54.1 |
|  |  | 95.8% | 85.3% | 47.0 | 95.2% | 84.7% | 46.9 | 95.6% | 81.6% | 45.0 | 93.7% | 87.3% | 48.5 |
| **SG18_197** | **CTR** | 90.0% | 81.2% | 50.1 | 89.9% | 80.0% | 49.4 | 92.0% | 78.5% | 47.4 | 90.2% | 84.8% | 51.5 |
|  |  | 91.4% | 82.7% | 47.8 | 91.5% | 81.7% | 47.1 | 93.2% | 80.0% | 45.3 | 91.5% | 86.3% | 49.2 |
|  |  | 87.9% | 79.2% | 39.8 | 88.3% | 78.6% | 39.4 | 90.8% | 77.4% | 37.7 | 89.0% | 83.5% | 41.0 |
|  | **SAL** | 94.4% | 84.4% | 53.2 | 93.7% | 83.6% | 53.0 | 94.4% | 80.3% | 50.6 | 92.6% | 86.6% | 54.9 |
|  |  | 93.8% | 83.4% | 47.9 | 93.3% | 83.1% | 47.8 | 94.1% | 80.1% | 45.7 | 91.7% | 85.4% | 49.3 |
|  |  | 94.2% | 84.7% | 49.8 | 93.7% | 83.8% | 49.5 | 94.3% | 80.7% | 47.3 | 92.8% | 87.2% | 51.3 |
| **SG18_247** | **CTR** | 94.1% | 84.4% | 54.0 | 93.7% | 83.7% | 53.7 | 94.1% | 80.5% | 51.5 | 90.9% | 85.1% | 55.6 |
|  |  | 93.3% | 83.5% | 53.8 | 93.0% | 82.9% | 53.5 | 93.3% | 79.5% | 51.1 | 90.3% | 84.5% | 55.3 |
|  |  | 89.9% | 81.0% | 48.9 | 90.3% | 80.2% | 48.2 | 91.5% | 77.8% | 46.2 | 89.3% | 83.5% | 50.1 |
|  | **SAL** | 93.9% | 83.6% | 51.9 | 93.6% | 83.2% | 51.7 | 94.2% | 80.2% | 49.6 | 92.0% | 85.7% | 53.4 |
|  |  | 94.5% | 84.4% | 49.4 | 94.2% | 84.0% | 49.3 | 94.8% | 81.0% | 47.3 | 92.6% | 86.6% | 51.0 |
|  |  | 94.2% | 83.8% | 47.0 | 93.9% | 83.5% | 46.9 | 94.4% | 80.2% | 44.9 | 92.3% | 86.0% | 48.5 |

**Table S2**. Primers used for RT-PCR.

| Oligo Name | Sequence (5' to 3') |
| --- | --- |
| EF_Solyc07g016150.4_For | CAGCTAAACTTGTTCCTGTTGG |
| EF_Solyc07g016150.4_Rev | GTCACAACTCTGAACGTACTCG |
| Actin_Solyc02g094320.3_For | AGGCTTGATTTTGAACTTGTACAG |
| [Actin_Solyc02g094320.3_Rev](javascript:__doPostBack('lbDescription_2112404','')) | TGCACCAGATTACGGAATAAGC |
| KCOA_Solyc03g005320.3_For | AGAAGGAGATTGGTGTTGCC |
| KCOA_Solyc03g005320.3_Rev | GGAGTTGCTCAGACATAGGAAG |
| CRF_Solyc01g008890.4_For | GCAGCCATTGAAATAAGAGGTG |
| CRF_Solyc01g008890.4_Rev | AGGTGGCACGATGAAGTTG |
| [PX_Solyc04g080760.3_For](javascript:__doPostBack('lbDescription_2112443','')) | TGTTGCATCACCAATCAGATTTG |
| [PX_Solyc04g080760.3_Rev](javascript:__doPostBack('lbDescription_2112443','')) | GCATAACTCTTCACCAATTCCTTG |
| [HKT12_Solyc07g014680.4_For](javascript:__doPostBack('lbDescription_2112440','')) | TTCTCATTCTTATCCCTCAAGTCC |
| [HKT12_Solyc07g014680.4_Rev](javascript:__doPostBack('lbDescription_2112440','')) | GTGAATATCCAACACATTTTGAGTTC |
| [APX2_Solyc09g007270.3_For](javascript:__doPostBack('lbDescription_2112404','')) | GGCACCCGAATGAACTTAAAC |
| [APX2_Solyc09g007270.3_Rev](javascript:__doPostBack('lbDescription_2112404','')) | TGATAAAAGTCAGCGTAGGATAGG |
| [GSTR_Solyc03g071850.1_For](javascript:__doPostBack('lbDescription_2112433','')) | GGAAGACATGGAAGAGATAGCC |
| [GSTR_Solyc03g071850.1_Rev](javascript:__doPostBack('lbDescription_2112433','')) | CCCTTCCCCTGTTTTCAATTC |
| [AQP_Solyc03g005980.3_For](javascript:__doPostBack('lbDescription_2112432','')) | CCAGGAGTAGCCATAACTTGG |
| [AQP_Solyc03g005980.3_Rev](javascript:__doPostBack('lbDescription_2112432','')) | CGAGAAAATACCTGTTTCCAAGG |
| CDPK_Solyc12g099790.3_For | AAAGAGGATATTGAGGATGTAAGGAG |
| CDPK_Solyc12g099790.3_Rev | AAGTTCACCTCCAGCACAC |


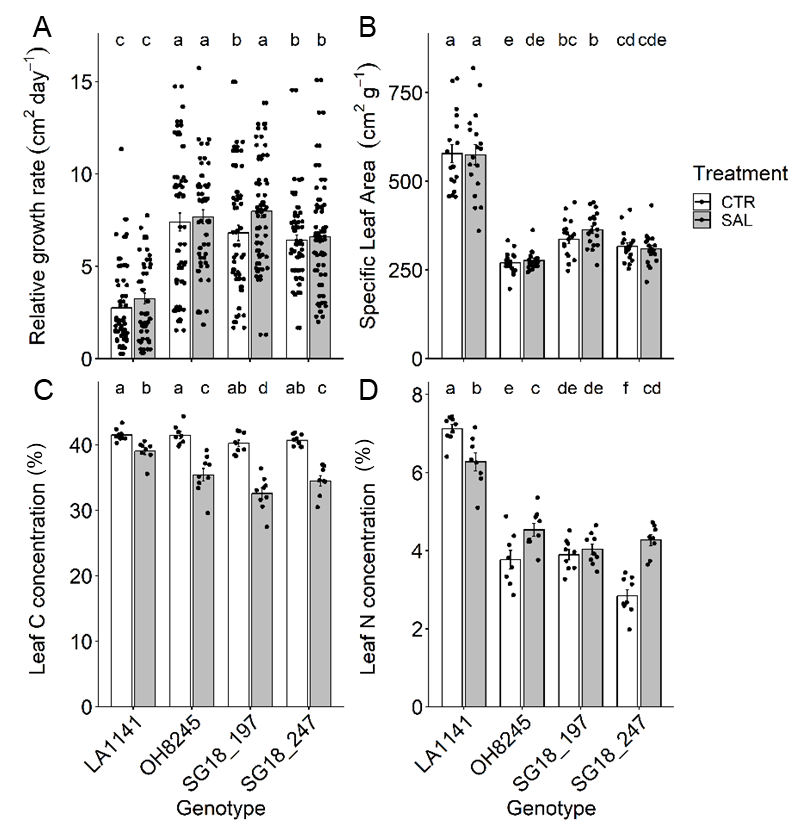


**Figure S2**. Plant relative growth rate (A) within four weeks and specific leaf area (B), leaf C (C) and N (D) concentration after two to three weeks of control (1.5 dS m^-1^, CTR) and salinity treatment (12 dS m^-1^, SAL) of the tomato wild relative (*Solanum galapagense*; LA1141), tomato OH8245, and two introgression lines derived from their crossing (SG18_197 and SG18_247). Data show mean ± standard error. Different letters indicate statistical significance (α =0.05).

**Table S3**. List of notable salinity tolerance traits identified in this study and associated genotypes and root molecular responses.

| Salinity tolerance trait | Genotype | Associated genes |
| --- | --- | --- |
| High *Lp*_r_ | LA1141 | *Solyc02g083510*, Aquaporin  *Solyc03g006810*, Peroxidase  *Solyc05g052360*, Laccase (lignin degradation)  *Solyc11g073140*, LEA protein-like protein |
| Lower RTD | LA1141  SG18_247 | *Solyc04g005620*, Casparian strip membrane protein  *Solyc10g083250*, Casparian strip membrane protein |
| Higher *P_n_* | SG18_247 | ? |
| Lower respiration over *P_n_* | LA1141  SG18_247 | *Solyc06g072510*, mitochondrial phosphate carrier |
| High leaf C | LA1141 | ? |
| High SLA | LA1141  SG18_197 | ? |
| Lower stomatal density | LA1141 | ? |
| Higher **Ψ**_stem_ | LA1141  SG18_247  SG18_197 | ? |
| High **Ψ_π_** | LA1141  SG18_247 | ? |
| Lower ABA | LA1141 | ? |
| High leaf N | LA1141 | *Solyc11g069750*, High-affinity nitrate transporter 2.2 |
| Low leaf Na^+^ | LA1141  SG18_247 | *Solyc06g008820*, Na^+^/H^+^ antiporter 1  *Solyc07g014690*, Na^+^ transporter |
| Low leaf Na^+^/K^+^ | LA1141  SG18_247 | *Solyc01g098190*, (Na^+^/K^+^)/proton exchanger 4 |
| High leaf Ca^2+^ | LA1141  SG18_197 | *Solyc07g006370*, Cation/Ca^2+^ exchanger 1 |
| High leaf Cu | LA1141 | *Solyc08g061610*, Cu-transporting ATPase PAA2 |


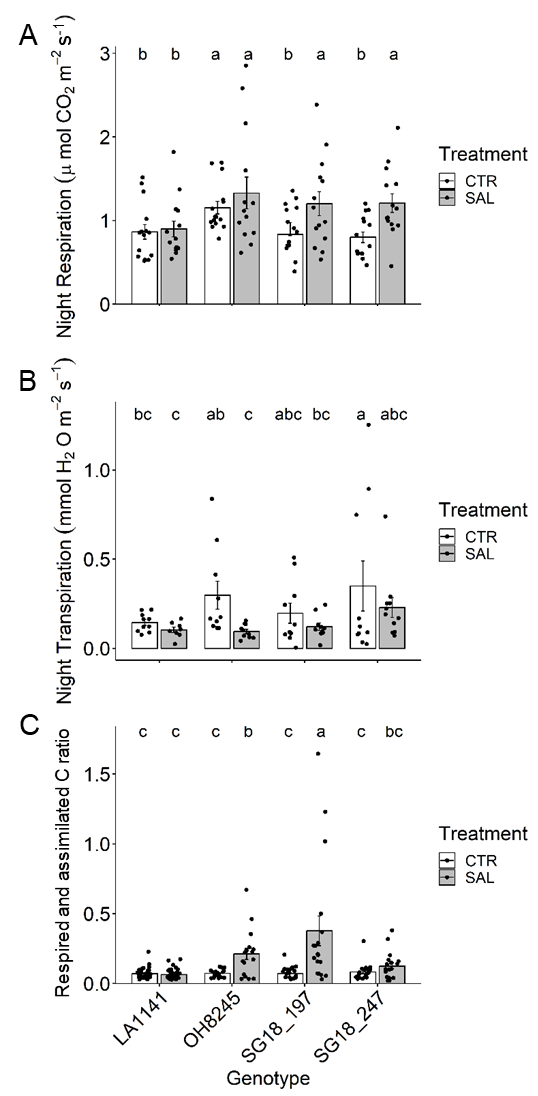
Figure S3. Night respiration (A), night transpiration (B) and ratio between respired and assimilated carbon obtained from leaf spot measurements (C) after two to three weeks of control (1.5 dS m^-1^, CTR) and salinity treatment (12 dS m^-1^, SAL) of the tomato wild relative (*Solanum galapagense*; LA1141), tomato OH8245, and two introgression lines derived from their crossing (SG18_197 and SG18_247). Data show mean ± standard error. Different letters indicate statistical significance (α =0.05).

**
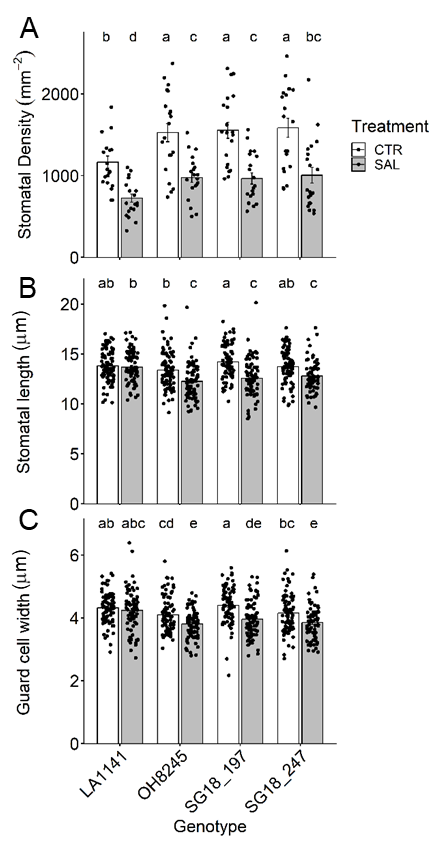
Figure S4**. Stomatal density (A), length (B), and guard cell width (C) within four weeks of control (1.5 dS m^-1^, CTR) and salinity treatment (12 dS m^-1^, SAL) of the tomato wild relative (*Solanum galapagense*; LA1141), tomato OH8245, and two introgression lines derived from their crossing (SG18_197 and SG18_247). Data show mean ± standard error. Different letters indicate statistical significance (α =0.05).


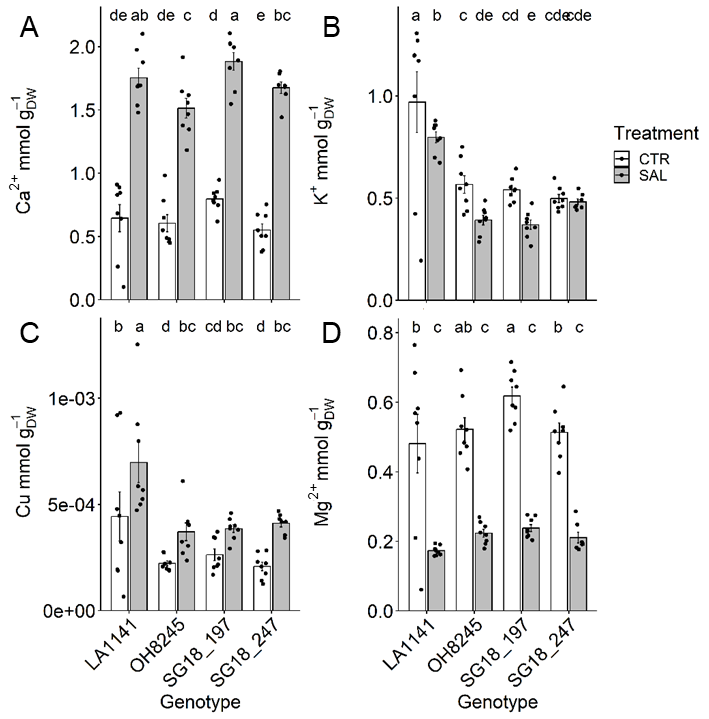


**Figure S5**. Leaf nutrient profile after three to four weeks of control (1.5 dS m^-1^, CTR) and salinity treatment (12 dS m^-1^, SAL) of the tomato wild relative (*Solanum galapagense*; LA1141), tomato OH8245, and two introgression lines derived from their crossing (SG18_197 and SG18_247). Data show mean ± standard error. Different letters indicate statistical significance (α =0.05).

**Table S4**. Library size and quality of each sample for RNA-seq.

| **Genotype** | **Treatment** | **% Proper Pairs** | **M Assigned** | **GC content** | **% Adapter** | **Million Seqs** |
| --- | --- | --- | --- | --- | --- | --- |
| **LA1141** | **CTR** | 93.80% | 47.4 | 42.70% | 18.00% | 56.5 |
|  |  | 92.20% | 51.9 | 42.80% | 19.20% | 61.4 |
|  |  | 88.60% | 45.0 | 43.40% | 18.40% | 57.3 |
|  | **SAL** | 92.20% | 50.2 | 42.80% | 19.20% | 59.5 |
|  |  | 93.30% | 50.3 | 42.80% | 17.40% | 59.5 |
|  |  | 93.10% | 40.0 | 42.80% | 16.70% | 46.9 |
| **OH8245** | **CTR** | 84.10% | 40.9 | 43.50% | 18.00% | 51.9 |
|  |  | 90.40% | 42.6 | 43.40% | 16.90% | 52.8 |
|  |  | 87.70% | 41.6 | 43.60% | 16.60% | 53.7 |
|  | **SAL** | 95.50% | 49.2 | 42.90% | 17.10% | 57.5 |
|  |  | 95.20% | 52.6 | 42.80% | 18.00% | 62.1 |
|  |  | 95.80% | 47.0 | 42.80% | 15.80% | 55.0 |
| **SG18_197** | **CTR** | 90.00% | 50.1 | 43.40% | 16.40% | 62.6 |
|  |  | 91.40% | 47.8 | 43.70% | 17.80% | 60.9 |
|  |  | 87.90% | 39.8 | 43.90% | 17.00% | 56.5 |
|  | **SAL** | 94.40% | 53.2 | 42.90% | 18.20% | 62.7 |
|  |  | 93.80% | 47.9 | 42.90% | 17.40% | 57.2 |
|  |  | 94.20% | 49.8 | 43.00% | 18.20% | 57.8 |
| **SG18_247** | **CTR** | 94.10% | 54.0 | 42.90% | 20.00% | 63.0 |
|  |  | 93.30% | 53.8 | 42.80% | 19.40% | 63.3 |
|  |  | 89.90% | 48.9 | 43.30% | 17.10% | 60.7 |
|  | **SAL** | 93.90% | 51.9 | 42.80% | 17.80% | 60.9 |
|  |  |  |  |  |  |  |
|  |  | 94.50% | 49.4 | 42.90% | 16.50% | 58.8 |
|  |  | 94.20% | 47.0 | 42.80% | 18.50% | 55.4 |


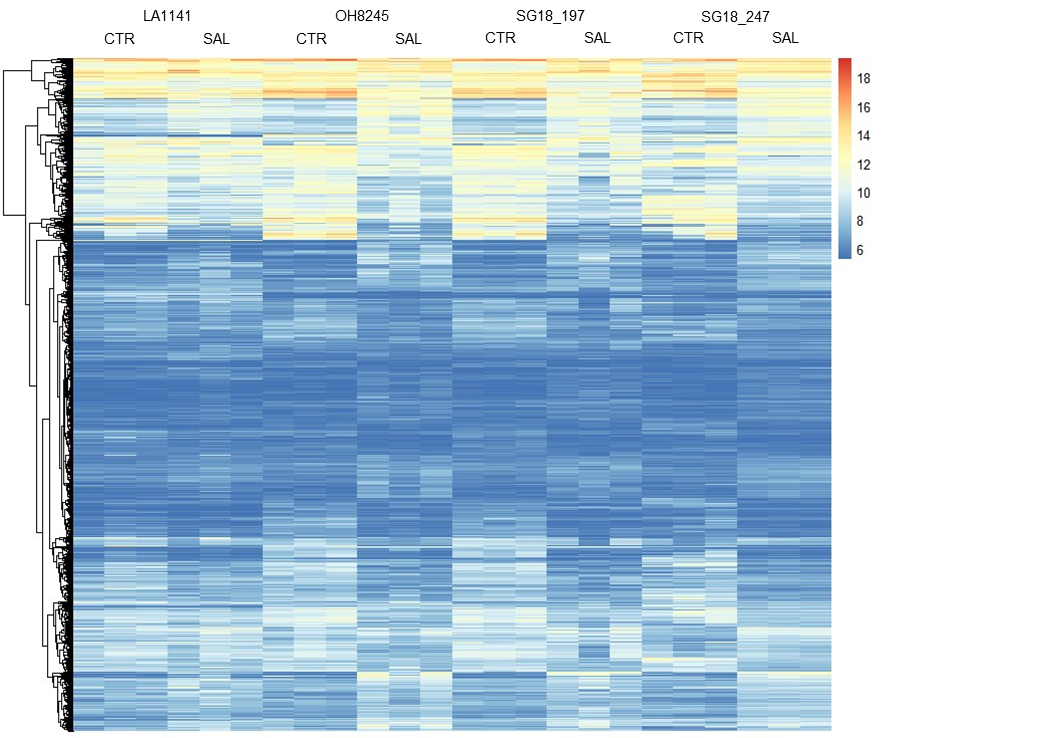


Figure S6. Heat map and hierarchical clustering of differentially expressed genes between LA1141, OH8245, SG18_197 and SG18_247 in response to three weeks of salinity treatments (12 dS m^-1^) compared to their controls (1.5 dS m^-1^). To select the differentially expressed genes, we set threshold as p value <0.05 and |log2FC| >2.


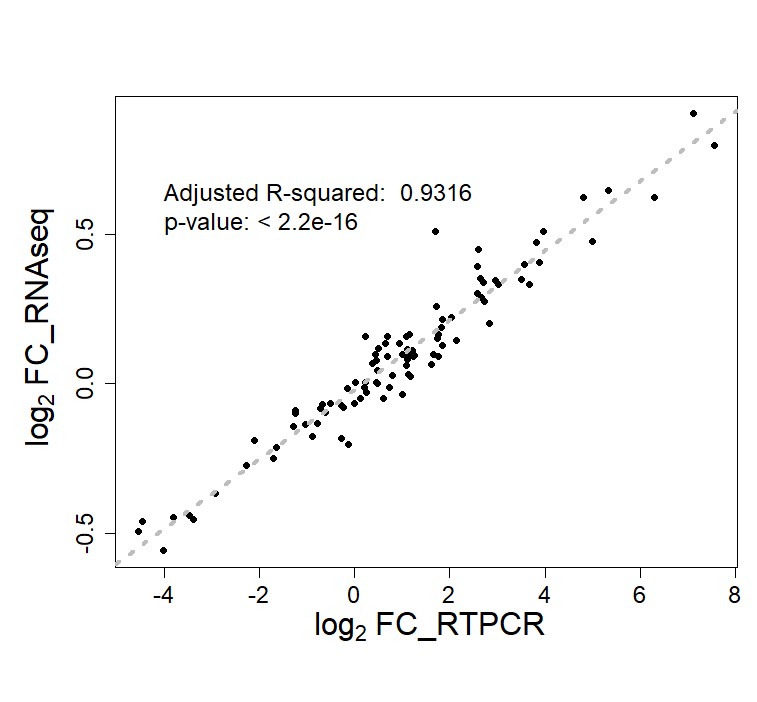


**Figure S7**. Relative expression data from RNA-seq and RT-PCR analyses of eight genes in LA1141, OH8245, SG18_197 and SG18_247 in response to three weeks of salinity treatment (12 dS m^-1^) compared to their controls (1.5 dS m^-1^).

Table S5. Enriched biological processes of differentially expressed genes between control and salinity treatment in each genotype, and respective p values when <0.05 (blue represents p values closer to 0.05 and red farther from 0.05).

Table S6. Enriched cellular components of differentially expressed genes between control and salinity treatment in each genotype, and respective adjusted p values when <0.05 (blue represents p values closer to 0.05 and red farther from 0.05).

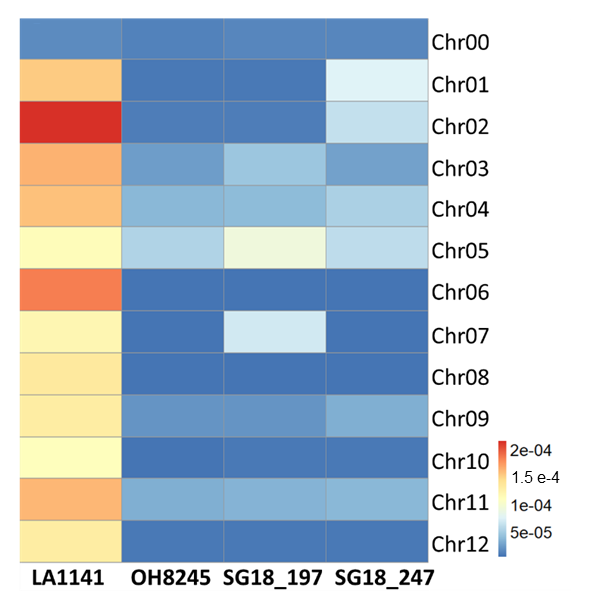


**Figure S8**. Transcriptome SNP density in each chromosome per genotype.

**Table S7**. Number of high-, moderate-, and low-impact variants classified based on SnpEff analysis.

| Genotype | High | Moderate | Low |
| --- | --- | --- | --- |
| LA1141 | 521 | 10685 | 10788 |
| OH8245 | 452 | 10535 | 10645 |
| SG18_197 | 459 | 10324 | 10419 |
| SG18_247 | 474 | 9564 | 9673 |

**Table S8.** Genes with high-impact variants in common between LA1141 and SG18_197 and SG_247 respectively.

| Introgression line | Gene | Functional annotation |
| --- | --- | --- |
| SG18_197 | Solyc01g005783 | Unknown protein |
|  | Solyc01g087400 | RING/FYVE/PHD-type zinc finger family protein |
|  | Solyc01g090210 | U-box domain-containing protein 35 |
|  | Solyc01g106330 | Glutamyl-tRNA(Gln) amidotransferase subunit A |
|  | Solyc01g107750 | phosphatidylinositol 3,4,5-trisphosphate 3-phosphatase and protein-tyrosine-phosphatase PTEN2A-like |
|  | Solyc02g084490 | Golgin candidate 6 |
|  | Solyc03g006520 | Splicing factor 3B subunit 2 |
|  | Solyc03g032130 | Unknown protein |
|  | Solyc03g119520 | BnaC05g48200D protein |
|  | Solyc06g074770 | U box domain |
|  | Solyc07g019630 | Unknown protein |
|  | Solyc07g055830 | Transcription elongation factor B polypeptide |
|  | Solyc08g008550 | Serine/threonine-protein kinase fray2 |
|  | Solyc08g060840 | Protein FAR-RED IMPAIRED RESPONSE 1 |
|  | Solyc08g074240 | 40S ribosomal protein S6 |
|  | Solyc09g075430 | Ribosomal protein L19 |
|  | Solyc10g007770 | Protein MEI2-like 5 |
|  | Solyc10g086190 | Adenosine kinase |
|  | Solyc11g066040 | RING-type E3 ubiquitin transferase |
| SG18_247 | Solyc01g010750 | Ricin B lectin domain |
|  | Solyc01g011413 | Unknown protein |
|  | Solyc01g079090 | Protoporphyrinogen oxidase |
|  | Solyc01g080540 | Histidine-containing phosphotransfer protein 1 |
|  | Solyc02g062120 | Membrane fusion protein Use1 |
|  | Solyc02g067240 | Unknown protein |
|  | Solyc02g077570 | MLO-like protein |
|  | Solyc02g084260 | DNA mismatch repair protein MLH3 |
|  | Solyc03g116900 | Metal transporter Nramp3 |
|  | Solyc03g117770 | Serinc-domain containing serine and sphingolipid biosynthesis protein |
|  | Solyc04g074167 | classical arabinogalactan protein 26-like |
|  | Solyc04g074880 | purine permease 3-like |
|  | Solyc04g078090 | Acyl-CoA-binding domain-containing protein 2 |
|  | Solyc05g026510 | Sister-chromatid cohesion protein 3 |
|  | Solyc06g083210 | Serine/threonine-protein kinase MHK |
|  | Solyc07g053250 | F-box protein isoform X1 |
|  | Solyc08g005910 | Ankyrin repeat |
|  | Solyc08g080270 | UDP-galactose/UDP-glucose transporter 2-like |
|  | Solyc09g064590 | Pre-mRNA-processing factor 39-like protein |
|  | Solyc09g075990 | Unknown protein |
|  | Solyc09g091480 | RING/U-box superfamily protein |
|  | Solyc09g150143 | Unknown protein |
|  | Solyc10g005510 | Glyceraldehyde-3-phosphate dehydrogenase |
|  | Solyc11g045310 | Transcription factor, MADS-box |
|  | Solyc12g094570 | inactive purple acid phosphatase-like protein |
